# The factors affecting household transmission dynamics and community compliance with Ebola control measures: a mixed-methods study in a rural village in Sierra Leone

**DOI:** 10.1186/s12889-018-5158-6

**Published:** 2018-02-13

**Authors:** Grazia Caleo, Jennifer Duncombe, Freya Jephcott, Kamalini Lokuge, Clair Mills, Evita Looijen, Fivi Theoharaki, Ronald Kremer, Karline Kleijer, James Squire, Manjo Lamin, Beverley Stringer, Helen A. Weiss, Daniel Culli, Gian Luca Di Tanna, Jane Greig

**Affiliations:** 1 Manson Unit, Médecins Sans Frontières (MSF), London, UK; 2 MSF, Amsterdam, The Netherlands; 3 Department of Veterinary Medicine, University of Cambridge, Cambridge, UK; 4 National Centre for Epidemiology and Population Health, Research School of Population Health, Australian National University, Canberra, Australia; 5 District Health Management Team, Ministry of Health and Sanitation, Kailahun, Sierra Leone; 6 MSF, London, UK; 7 MRC Tropical Epidemiology Group, Faculty of Epidemiology and Population Health, London School of Hygiene and Tropical Medicine, London, UK; 8 Centre for Primary Care and Public Health, Queen Mary University of London, London, UK

**Keywords:** Ebola virus disease, Transmission dynamics, Community perception

## Abstract

**Background:**

Little is understood of Ebola virus disease (EVD) transmission dynamics and community compliance with control measures over time. Understanding these interactions is essential if interventions are to be effective in future outbreaks. We conducted a mixed-methods study to explore these factors in a rural village that experienced sustained EVD transmission in Kailahun District, Sierra Leone.

**Methods:**

We reconstructed transmission dynamics using a cross-sectional survey conducted in April 2015, and cross-referenced our results with surveillance, burial, and Ebola Management Centre (EMC) data. Factors associated with EVD transmission were assessed with Cox proportional hazards regression. Following the survey, qualitative semi-structured interviews explored views of community informants and households.

**Results:**

All households (*n* = 240; 1161 individuals) participated in the survey. 29 of 31 EVD probable/confirmed cases died (93·5% case fatality rate); six deaths (20·6%) had been missed by other surveillance systems. Transmission over five generations lasted 16 weeks. Although most households had ≤5 members there was a significant increase in risk of Ebola in households with > 5 members. Risk of EVD was also associated with older age. Cases were spatially clustered; all occurred in 15 households.

EVD transmission was better understood when the community experience started to concord with public health messages being given. Perceptions of contact tracing changed from invading privacy and selling people to ensuring community safety. Burials in plastic bags, without female attendants or prayer, were perceived as dishonourable. Further reasons for low compliance were low EMC survival rates, family perceptions of a moral duty to provide care to relatives, poor communication with the EMC, and loss of livelihoods due to quarantine. Compliance with response measures increased only after the second generation, coinciding with the implementation of restrictive by-laws, return of the first survivor, reduced contact with dead bodies, and admission of patients to the EMC.

**Conclusions:**

Transmission occurred primarily in a few large households, with prolonged transmission and a high death toll. Return of a survivor to the village and more effective implementation of control strategies coincided with increased compliance to control measures, with few subsequent cases. We propose key recommendations for management of EVD outbreaks based on this experience.

## Background

The first case of Ebola virus disease (EVD) in Sierra Leone is believed to have occurred in mid-May 2014, in a remote village of Kailahun District (estimated population 465,048) [1, 2]. On 12th June 2014, the President of Sierra Leone declared a state of emergency in the district [3]. The last case was recorded in Kailahun in mid-December 2014 and the Ministry of Health and Sanitation (MoHS) declared Kailahun District free from human-to-human transmission on 22nd January 2015, following 42 continuous days without a confirmed case [1]. Médecins sans Frontières (MSF) opened an Ebola Management Centre (EMC) in Kailahun on 26th June 2014 to support the district MoHS [4]. The MSF EMC was the only functioning Ebola management centre in the district, responsible for isolating 63·0% of confirmed cases. In total, the district MoHS reported 565 confirmed EVD cases in the population of Kailahun (attack rate 0·12%), including 287 deaths (case fatality rate [CFR] 51·0%) [5].

Evidence-based interventions for EVD control include early detection of cases through effective surveillance and contact tracing, admission of symptomatic cases to EMCs where staff adhere to high standards of infection control procedures, and safe burials by trained teams [6, 7]. Quarantine measures were also widely implemented [8], and by-laws imposed that included travel restrictions and penalties for hiding suspected cases [9].

The transmission dynamics of the West Africa EVD epidemic have, so far, been reconstructed from EMC and surveillance data, and mathematical modelling [4, 10–12]. However, poor surveillance systems and limited EMC capacities are likely to have resulted in underestimation of the true extent of the outbreak, limiting the ability to understand the dynamics and experience of the epidemic at community level, in particular in Sierra Leone, the country most affected by the West Africa EVD outbreak [13, 14].

Little is known of the factors that influence EVD transmission dynamics and community compliance with control measures over time. Such understanding is essential if interventions are to be effective, particularly in areas like Sierra Leone with no previous local EVD experience. In order to address this knowledge gap and inform future responses, we conducted an in-depth mixed-methods study in a rural village in Kailahun District that experienced prolonged EVD transmission during the outbreak.

## Methods

To enable assessment of behaviour adaptation over time, we used data from MSF EMC patient registers to select a village in the district that had experienced a very protracted EVD outbreak. We then conducted a mixed-methods study combining data gathered via a cross-sectional survey and semi-structured interviews in this selected village. The cross-sectional survey data were used to reconstruct the dynamics of transmission. Semi-structured interviews were used to document community perception, resistance, and adaptation to response strategies. Survey and interview data were triangulated with data from the safe burial and MoHS surveillance databases to verify the reconstruction of the EVD transmission, and explain changes in transmission and behaviour over time.

### Cross-sectional survey

All consenting households in the village were included in the cross-sectional household survey. A trained MSF team, using a validated instrument for household mortality studies and EVD case investigation forms, collected demographic data from household heads on household members, births, arrivals, departures, deaths, illnesses (including signs and symptoms compatible with the EVD case definition), and history of contact with individuals symptomatic for EVD [15, 16]. Verbal consent for participation was obtained from the head of each household after a briefing about the aim of the survey, the questions and duration of the questionnaire, and the option to end the interview at any time if wished.

The household survey was conducted in April 2015, with a recall period for responses between May 2014 (date of the first reported EVD case in the district) and the date of the survey. A local events calendar was developed to aid recall. MSF-EMC patient registers were used to verify the date of admission, symptoms, laboratory confirmation of EVD, and outcomes of patients admitted to the EMC. Each household in the village was enumerated and listed; from this list we randomly selected the households for the semi-structured interview.

Geographic positioning system (GPS) data were used to map the village layout and location of all households. Data were de-identified and entered into a password-protected electronic database.

### Semi-structured interviews

At the end of the cross-sectional survey*,* semi-structured interviews were conducted with key community informants and selected households. Households were divided into two groups based on whether they had experienced at least one EVD case or no EVD cases. Ten households were randomly selected for interview from each group (total of 20 interviews).

A purposive approach was used to select key community informants: traditional healers; biomedical health-care providers; and community leaders including tribal authorities, heads of community groups, and religious leaders. The heads of the selected households and key community informants were interviewed after verbal consent to participate was obtained. Participants were briefed about study objectives, questions and duration of interview, and the option to leave the study at any time. All interviews were semi-structured, took place in a private space, and were conducted by a trained MSF team.

Interviews were conducted in the local language using an interpreter to translate and back translate to English. The local events calendar developed for the household survey was also used in the semi-structured interviews. Topic guides directed interviewers to explore changes over time in perceptions of EVD and perspectives related to EVD response activities including contact tracing activities, the MSF EMC, the safe burial team, and quarantine. Interviews explored how these EVD control strategies were implemented and how these accorded with cultural beliefs. The topic guide was the same for household and key informant groups except for an additional section in the key informant guide, regarding how the outbreak started in the village. After initial data analysis had been completed, a summary narrative was compiled and shared with the village in the format of a story. Participant validation was achieved in this way in order to refine findings [17].

### Case definitions

World Health Organization (WHO) EVD case definitions were used to define suspect, probable, and confirmed cases [16]. A suspect case was defined as: any person, alive or dead, suffering or having suffered from sudden onset of high fever and having had contact with a suspect, probable, or confirmed EVD case or with a dead or sick animal; any person with sudden onset of high fever and at least three relevant symptoms (headaches, vomiting, anorexia/loss of appetite, diarrhoea, lethargy, stomach pain, aching muscles or joints, difficulty swallowing, breathing difficulties, hiccup); any person with inexplicable bleeding; or any sudden, inexplicable death. A confirmed case was defined as anyone with a positive quantitative reverse transcription polymerase chain reaction (PCR) result. PCR cycle threshold (Ct) results were used as indicators of viral load. The lower the Ct value the higher the viral load [18]. A probable EVD case was defined as anyone who met the clinical case definition and had a history of contact with a person with confirmed EVD, but who did not have a confirmed laboratory test result [16].

### Data analysis

Cox proportional hazards regression models were fitted to estimate hazard ratios (HRs) and 95% confidence intervals (95% CI) for the association between EVD (probable and confirmed cases) and covariates previously documented to be associated with EVD, including household size, sex, and age [19, 20]. Events were dated by epidemiological week and used as the time parameter in the Cox model. Cox shared frailty models were used to allow for within-household correlation.

The crude mortality rate (CMR) and EVD-specific mortality rate were estimated as deaths during study period/(mid-period population at risk x duration of period), where mid-period population at risk accounted for births, deaths, arrivals, and departures during the recall period [21]. Mortality rates were expressed as deaths per 10,000 per day. The attributable risk percent (AR%) and population attributable risk percent (PAR%) were used to estimate excess mortality risk due to EVD in the exposed households and village level, respectively.

The proportion of EVD cases isolated by admission to the EMC and the proportion of people who died from EVD and received safe burial were assessed by comparing cases reported in MoHS surveillance, EMC, and burial team data with cases (confirmed and probable) identified through the household survey.

Transmission dynamics were constructed using contact history, and described using transmission chains. Relationships between individuals were categorised as nuclear (immediate family), extra nuclear (extended family), and social (neighbours and friends).

Statistical analyses were carried out using Stata 14.0 (Stata Corporation, Texas-USA); maps were generated using QGIS™ software (version 2.14, https://qgis.org/en/site/). Participant responses from all semi-structured interviews were translated and transcribed at the time of the interview. Key community informants and household interview data were analysed separately using an inductive framework approach via an iterative process of coding and categorization (using ©NVivo 10) leading to the identification of emerging themes. The former contributed to the description of initial phase of the outbreak along with documenting the village experience over time, and the latter to exploring affected and unaffected household experience.

## Results

### Study population

The village consisted of 240 households (1161 individuals); all heads of households gave consent to participate. The median age of villagers was 18 years (interquartile range [IQR] 7–34 years), with 44·4% (*n* = 515) younger than 15 years old. Approximately half the villagers were female (52·7%). Household size ranged from 1 to 17 people, with a median size of 5 (IQR 3–6).

### Transmission dynamics

Overall, 31 EVD cases (15 confirmed, 16 probable) were identified, giving an overall attack rate in the village of 2·7%. The index case was an adult male who was resident in a city that was a known EVD hotspot in June–July 2014. In late July 2014, while symptomatic, he travelled back to his village of origin and died 1 week after his return. Table 1 details the possible routes of EVD transmission that were reported by his household and key informants. There was no record of the index case being tested for EVD, although he was reportedly taken to a holding centre for testing.

**Table 1:** Possible sources of infection for the index case

Contact with EVD patient(s) in the course of his work as a pharmacist	
Contact with EVD patient(s) when he had a tooth extracted at a local Government Hospital, which was, at that time, a major hotspot of EVD	
Contact with a traditional healer, who reportedly came from Guinea to treat the index case, who may have been infected	
Contact with EVD patient(s) at a holding centre he was taken to for testing because he was symptomatic following the tooth extraction	
Unknown source of infection (e.g. community)	

**Fig. 1:**
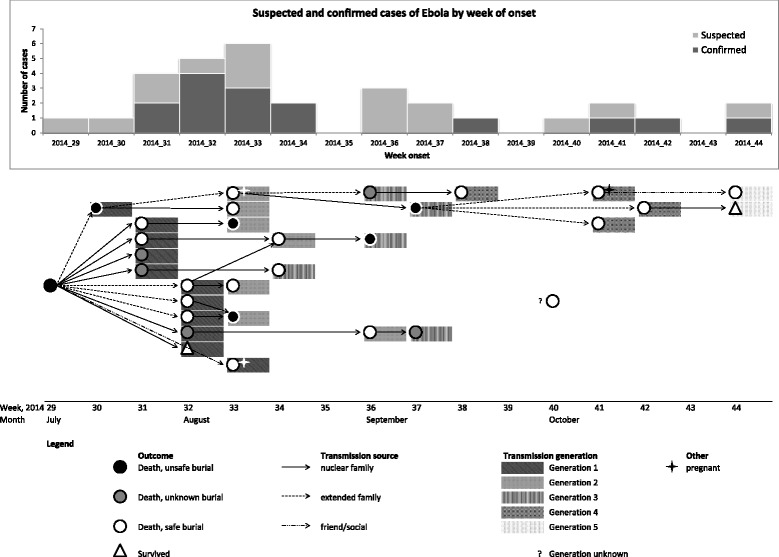
EVD transmission generation, according to week of onset

Following death, the index case was buried in an unsafe manner by community members, many of whom had unprotected contact with the body. It is believed that this may have started the chain of person-to-person transmission in the village. Transmission lasted for 16 weeks, with 30 cases arising over five transmission generations: 11 cases in the 1st generation, seven in the 2nd, five in the 3rd, four in the 4th, and two in the 5th. For the one remaining case, a traditional birth attendant, a clear source of infection and transmission generation was not established (Fig. 1). The time from exposure to symptom onset was ≤2 weeks for all cases with known exposure. The first survivor came back to the village in week 35 (late August), after 7 weeks of transmission, when most of the cases in the village had already occurred.

Amongst the secondary cases with known exposure: 38·0% (11/29) had, as sole exposure, contact with a symptomatic person who was a probable/confirmed case; 10·3% (3/29) had a history of attending a funeral; and almost half (14/29; 48·2%) had history of both contact with a symptomatic person and a funeral exposure. The proportion of cases exposed via a funeral decreased over time from 90·9% (10/11) in the 1st generation to 71·4% (5/7) in the 2nd, 40·0% (2/5) in the 3rd, 25·0% (1/4) in the 4th, and none in the last. Contact with a symptomatic person increased from 72·7% (8/11) in the first to 100·0% in the following generations. Among the 30 secondary cases, 28 died (93·3%) and two survived (6·7%).

**Fig. 2:**
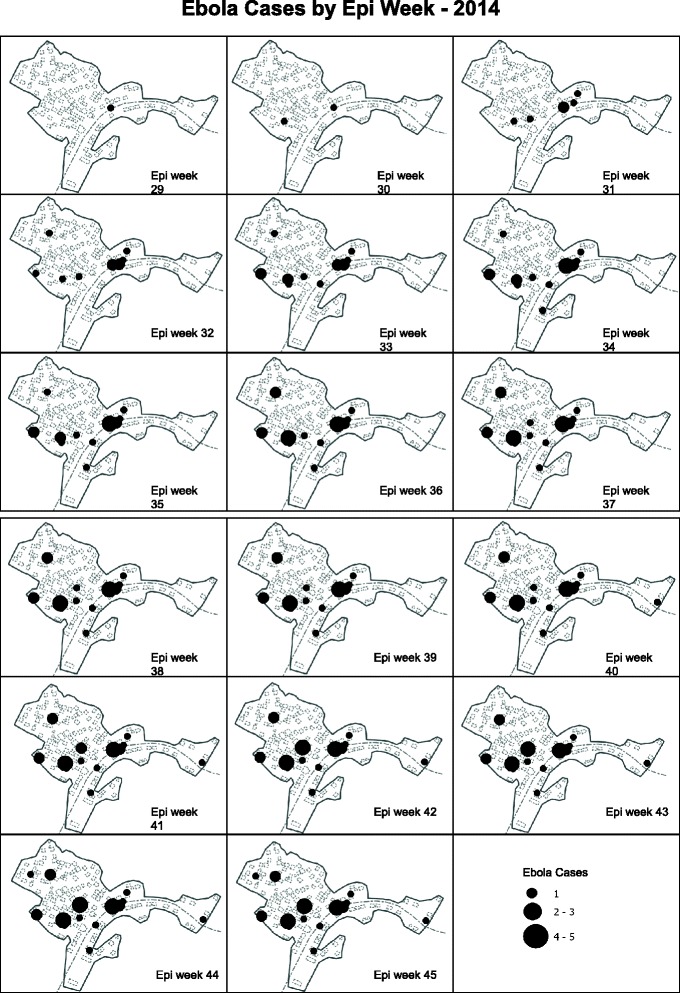
Geographical distribution of cases over time, weeks 29 –week 45

There was strong evidence of clustering of EVD (*p* < 0·0001), with all cases occurring in 15 of the 240 households (Fig. 2). Thirty-two percent of cases occurred in two households, in which cases occurred over three- and four-generation chains.

Most secondary cases were exposed via the nuclear (57·6%; 17/30) or extended family (30·0%; 9/30). Affected households had a median of seven members (IQR 6–8), and non-affected households a median of three (IQR 2–4) (*p* < 0·0001).

### Factors associated with EVD

EVD was associated with older age and household size in unadjusted analysis; these associations became stronger after adjustment for both variables and sex (Table 2). The rate of EVD was similar by sex (aHR 1·03; 95% CI 0·49–2·17 for females vs males), but was greater among those aged 15–54 years (aHR 23·04; 95% CI 3·06–173·12) and ≥55 years (aHR 57·28; 95% CI 7·03–466·33) compared with those aged 5–14 years, and among those living in larger (> 5 members) (aHR 56·53; 95% CI 19·64–162·73) compared with smaller households (Table 2).

**Table 2:** Demographic characteristics of the study participants and risk factors for EVD

	Entire village N	EVD infected N (% of village)	Crude hazard ratio	95% CI	p	Adjusted hazard ratio	95% CI	p	Adjusted hazard ratio from shared frailty Cox	95% CI	p
Sex											
Male	549	13 (2·4%)	ref			ref			ref		
Female	612	18 (2·9%)	1·24	0·61–2·54	0·55	1·03	0·49–2·17	0·92	1·19	0·52–2·73	0·68
Age group (years)											
< 5	174	4 (2·3%)	7·87	0·88–70·48	0·0001	6·02	0·66–54·39	< 0·0001	6·10	0·63–58·63	0·12
5–14	341	1 (0·3%)	ref			ref			ref		
15–54	552	18 (3·3%)	11·27	1·50–84·45		23·04	3·06–173·12		20·26	2·48–165·09	0·005
≥ 55	94	8 (8·5%)	31·57	3·94–252·48		57·28	7·03–466·33		53·06	5·89–477·66	< 0·0001
Household size											
≤ 5 members	973	4 (0·4%)	ref			ref			ref		
> 5 members	188	27 (14·4%)	37·15	12·99–106·19	< 0·0001	56·53	19·64–162·73	< 0·0001	56·08	16·38–191·92	< 0·0001

### Mortality

Of the 31 cases (index case plus 30 secondary cases), 29 died (CFR 93·5%; 95% CI 78·6–99·2%). Thirteen of 15 confirmed cases and all 16 probable cases died. About half (55·2%) of EVD deaths were among females; three were pregnant and miscarried at home.

The community reported five non-EVD deaths during the recall period. The CMR for all causes of death (EVD and other) was 0·97 per 10,000 per day. EVD-specific CMR was 0·83 per 10,000 per day and the non-EVD CMR was 0·14 per 10,000 per day.

The AR% for death associated with EVD was 99·5% (95% CI 98·6–99·8) among the exposed households, while the PAR% for death associated with EVD in the whole village was 84·5%.

### Admission to the MSF EMC

In mid-August 2014, cases started to be admitted to the EMC. Of the 31 cases, 15 were admitted to the EMC and had Ebola infection confirmed by PCR testing. Twelve cases had an exact date of symptom onset recorded, with a median time from first onset of symptoms to admission of 4·0 days (IQR 3–5). The median time to admission was 5·0 days in the first generation (IQR 4–7), falling to 1·0 day in the last generation (IQR 0–1). The mean Ct value at admission was 21·8 (SD 4·5). Among the confirmed cases at EMC, 12 (80·0%) presented at admission with at least one wet symptom (diarrhoea, vomiting, or bleeding).

### Burial, quarantine, and contact tracing

Of the 29 EVD deaths, 13 (44·8%) occurred within the EMC; five deaths in the community then had a safe burial by the burial team. Six deaths (20·6%) were captured during the survey but were not listed in the EMC, MoHS surveillance system and/or safe burial database. A further five people who died were reported by families to have been transported to an MSF or local Government hospital, however, there was no record of those patients in the EMC database. Contact tracing was reported to have occurred starting in late July; one in five village households reported they had been under contact tracing and quarantine measures. However, in August 2014, when 18 secondary cases had already occurred, the entire village was put under restriction of movements.

### Community perception, resistance, and adaptation to response activities

Semi-structured interviews were conducted with 38 participants: 10 households reporting EVD cases (affected households (AH)), 10 households with no cases (UH), and 18 key community informants (CI).

### Introduction of EVD in the village

When discussing how EVD had been introduced to the village, all participants referred to a single member or index case in the family or community, ranging from a family visitor to a health worker.

“The man [index case] brought Ebola here. He used to treat people in [city] that was a hotspot at the time. When he got sick, he came here to see traditional healers and a herbalist came from Guinea to treat him using traditional herbs.” – (CI09_m)

“An ambulance came to collect him and take him to [XX] holding centre. It was anecdotally reported that he tested negative, so some relatives went to pick him up. People were very happy, so they came to greet him/celebrate.” *-* (CI04_m)

### Misgivings toward Ebola

Initially, it was difficult for villagers to believe that infection could spread through everyday person-to-person contact. This perception was compounded by a climate of mistrust of authorities, fear of death, and lack of understanding of complex health messages such as the importance of isolation of those infected.

“We had never seen a sickness like this before, where you touch someone and you die.” - (CI12_m)

“It seemed like someone had poisoned our village; many, many, many people died. It was similar to other diseases [e.g. malaria].” – (CI16_m)

“We thought it was a curse; some people thought that it was some kind of traditional medicine that was being thrown on them.” - (CI13_f)

“People thought it was a conspiracy between the President and the westerners, who needed blood. They thought that if you go to the EMC, you will die.” - (CI03_m)

“People didn’t believe it: like war, we didn’t believe it could come here. There was lots of arguing - some people thought Ebola wasn’t real. They thought it was something sent by God.” - (CI04_m)

“People were hiding symptoms and deaths because they were scared of the camp [EMC]; by the time they were found and the ambulance called, they were already dead.” - (CI11_m)

“Early on, people were hiding if they were sick. By the time we knew they were sick, they weren’t alive long enough to send them to the EMC (1-2 days).” - (UH05_m)

“We beat the contact tracers - we thought they were responsible for our relatives’ deaths because they went for training at the same time [end of July] XX [index case] got sick.” - (CI16_m)

“At the start, people hated the contact tracers - they beat them. One man in particular was beaten almost to death.” - (CI17_f)

“The man [index case] came with a letter that said he should be isolated for 21 days. But we didn't understand what ‘isolation’ meant.” - (CI16_m)

### Change in perception

The perception of EVD held by the villagers changed when information received from contact tracers and the MSF health promotion team was consistent with what villagers observed in their lives at the community level. Implementation of the by-laws on travel and penalties for not reporting cases supported the understanding of the severity of the outbreak by villagers, and helped them accept that control measures were intended to protect and help the community.

“When we saw that people touched sick people and got sick, we could see the communication of it and realised that it is real.” - (CI13_f)

“Sensitisation from different sources [MSF/MoHS/radio] started to make sense; symptoms in our loved ones were exactly the same as they were telling us.” - (CI11_m)

“We realised that no contact was good, after a while, we saw the benefit.” - (CI15_m)

“But we had to follow the law we had to pay 500,000 Leones if there was a sick person found in the house.” - (CI12_m)

“It was for our own safety - to avoid touching bodies. To help them to stop the spread of Ebola. The word ‘safe’ equals ‘help’.” - (CI16_m)

### Behaviour adaptation

Understanding of the route of transmission, and observing survival of cases admitted to the EMC supported changes in behaviour and adaptation by the community. This mainly occurred in late August coinciding with the return of a survivor, reduced contact with dead bodies, restriction of movements and isolation of patients.

“When we heard about people surviving people's attitude changed.” - (CI03_m)

“We would go far away from the person and inform contact tracers who will call an ambulance to remove them to the camp [EMC].” - (UH04_f)

“Initially, it [burial team] was not good but when we saw that the deaths increased, we knew it was for our own safety.” - (AH02_m)

The village implemented a number of local measures to prevent spread between households.

“During the outbreak, some people even devised their own preventive measures, like stopping children from playing football so they don’t have contact with each other, and stop visit other households.” - (CI09_m)

“Traditional birth attendants stop doing deliveries.” - (CI17_f)

### Understanding control strategies and constraints

All strategies such us MSF/EMC, MSF health promotion, contact tracing, burial practices, quarantine/restriction of movements were understood by the community as helping to control EVD. However, resistance to specific practices that were perceived as offensive to socio-cultural norms was reported; this resistance continued until the value of such practices was understood.

### MSF/EMC

The EMC was understood to help people survive:

“Without the camp [EMC] - we would have no survivors.” - (CI04_m)

However, communication regarding the status of admitted patients was perceived as poor:

“We received no information while they were still alive. When they died, a nurse who worked at the camp [EMC] told us.” - (CI14_f)

“When the ambulance went with XX to the camp [EMC], some family members went to visit and they learned that he had died.” - (CI07_m)

The MSF health promotion team were perceived as empowering the community:

“It gave the Community Health Workers a zeal to call ambulances; they empowered us. They sensitised us about preventive methods and no touch.” - (CI06_m)

“Helped to decrease cases.” - (CI12_m)

“We learned not to touch other people, and use water and soap.” - (CI15_m)

### Contact tracing

Contact tracing was perceived as a mechanism to remove people from the community who were thought to be a risk, which initially created mistrust. This gave contact tracers a reputation for invading privacy and disrupting family and community life and sending people to their deaths.

“There was no sensitisation about why contact tracers were here. They would just call the ambulance and collect people to the EMC.” - (CI01_m)

“We didn’t like the contact tracers; called them murderers.” - (CI02_f)

“Invasion of privacy - it was not their business to investigate our household.” - (CI04_m)

“We didn’t like the fact that they were involving themselves in our affairs, we thought contact tracers were selling us to other people and that they were too inquisitive.” - (CI17_f)

However, contact tracers were valued once people understood that they were trying to protect people and prevent the spread of Ebola:

“It is our culture to touch people when they are sick, so if you don’t take people out of the village, people will touch them.” - (CI11_m)

“Without contact tracers we would have continued touching people. Instead, sick people were collected to the camp [EMC].” - (CI16_m)

“Otherwise we would have far more deaths.” - (CI14_f)

“Contact tracers should be empowered with training to stop the spread.” - (CI13_f)

### Burial practices

The value of safe burials was understood:

“Without the burial team, the disease would have spread because touching dead bodies is bad.” - (CI02_f)

However, burials were initially seen as lacking honour in terms of how they were performed, specifically the use of plastic bags, and the lack of burial clothes and prayers. Respondents also commented on the lack of women in the burial team and on the arrival of the teams in the village already dressed in personal protective equipment (PPE).

“Plastic bags are not traditional - there is no honour when you bury people this way.” - (CI03_m)

“Praying was not allowed.” - (CI09_m)

“Sometimes, in dreams, my husband appears and says ‘I have no clothes’.” - (AH06_f)

“Men burying women is not good; women should be part of the burial team.” - (CI17_f)

“We weren't happy about it. Before the outbreak, if a chief dies or a special person dies, they are buried by other special people. Now, we can't do that. There is no clothes, no dressing - and men are burying women, which is a problem for us.” - (CI11_m)

“People were afraid of the burial team when they came dressed in full protective clothes. They thought they were ghosts.” - (CI03_m)

In October, the burial procedures were improved to incorporate greater respect for local tradition:

“We couldn’t pray before, either, but now we can.” - (CI03_m)

“Now they [burial team] dress in protective clothes in the village.” - (CI11_m)

### Quarantine/restriction of movements

The community understood the value of quarantine:

“Because of quarantine, we couldn't spread Ebola to other households.” - (AH07_m)

However, people were also angry about quarantine:

“It destroyed many things, especially farming, our crops were destroyed and there is no food available now.” – (CI15_m)

In September, quarantine measures were improved by incorporation of food supply to quarantined households:

“We had no food at the start. They should have given us food like they did in other households at the end.” - (AH06_f)

### Affected versus unaffected households

Both affected and unaffected households were sensitive to law enforcement and were in favour of stricter methods to control Ebola in the future. The consequences of quarantine, in terms of financial and emotional impact and stigma, were harsher in affected households compared with non-affected households, since non-affected households were only directly impacted when the entire village was quarantined.

“Seven members of my family were taken to EMC. They all died there. Everyone would yell at us, ‘you brought Ebola here!’ I didn't - my brother did. But I still felt guilty.” - (AH03_m)

Affected households provide some insight into factors that led to continued transmission in some homes but not in others, and why within-household transmission continued even when between-household transmission was reducing:

“We could not abandon sick people – we must care for [them].” - (AH05_f)

“People didn’t come around - it was like the devil was here.” - (AH04_m)

## Discussion

Our study provides a comprehensive description of EVD in one village in Kailahun District, Sierra Leone that experienced sustained EVD transmission during 2014. We attempt to capture the complexities of the social context influencing outbreak control in this specific epidemic. We documented that immediate family members of large households were at greater risk of being infected, and because of the larger number of inhabitants, these households were more likely to maintain transmission. This finding corroborates insights from other studies. This may imply that future responses to an EVD outbreak could justify prioritization of affected large households and their immediate family members, in particular when human resources are insufficient to address the scale of the outbreak [19, 22].

Within affected households, transmission was maintained by the need to provide care for sick relatives, with cases continuing to occur over several generations. Compliance with response measures increased only after the second generation, coinciding with the return of a survivor, and strict implementation of other components of the EVD response, such as restriction of movements, reduced contact with dead bodies, and isolation of cases. However, this changing context only occurred after 7 weeks of transmission, when most of the cases in this outbreak had already died.

In particular, return of survivors to the village after treatment prompted a shift toward belief in Ebola and increasing acceptance of control measures. Late return of survivors prevented teams from building trust within the community. At the time that survivors returned, the village was experiencing a peak in case numbers, the MSF EMC was reaching the limit of its capacity (100 beds), and communication with households was primarily to inform of deceased loved ones, thus contributing to community fear and despair. People reported avoiding the MSF EMC because of poor survival rates, which reinforced the community perception of the EMC as a place where people die. One approach to improving community understanding and uptake of EMC services in future could include developing the role of an EMC-village liaison, whose role would be to support timely communication with communities about the status of relatives throughout admission. Use of EMC-village liaisons could acknowledge the gap in understanding of health system workers as to why patients may undermine control measures when faced with the need to look after their loved ones. Contact tracers could potentially play this liaison role, and therefore have the potential to be seen as providing something positive to the community rather than just reporting and tracing cases.

Reduced misgivings and doubt about Ebola were crucial to influencing attitudes toward control measures. This change likely occurred once the health messages given to the community mirrored their reality. Once Ebola transmission was understood, the perceptions of contract tracing changed from invading privacy, selling people, to working collectively toward community safety. The community then participated in control measures by setting up a number of local strategies such as stopping babies being delivered in the community, preventing children from playing contact games together, and not visiting other households. These strategies contributed to outbreak control, as observed by other authors [23]. Our findings emphasised the importance of the community having a role in tailoring outbreak responses. Following a localised governance approach may permit incorporation of accepted local social norms from the outset of intervention efforts, making them more acceptable and therefore effective.

Clear communication of complex health messages was challenging, but played a role in the acceptance of EVD control measures. It was essential that the community understood there was a 21-day incubation period, the importance of EMC isolation (both self-imposed and institutional), and that a single negative test result could not rule out disease during the incubation period. Other authors described similar issues for messaging in Sierra Leone and in previous outbreaks [24, 25].

Similar to the rest of the country, the age structure of the village was young, with those under age 15 accounting 44% of the population. The limited life experience of youth, and particularly collective experience with death from exposure to body fluids (e.g. “touch someone and you die”) or with infection prevention and control concepts (e.g. “we didn't understand what 'isolation' meant”) may have contributed to delays in understanding and adoption of the necessary responses, rather than villagers being deliberately uncooperative. However, we documented that regardless of age, the population in general suffered an overwhelming level of inexperience toward this disease and its impact. Response agencies must acknowledge community demographic structure and perspectives on the presence of EVD in parallel with launching control measures cognisant of their baseline understanding.

Our study findings show nuanced perceptions toward quarantine as both a way to control the spread of Ebola and a cause of social and livelihood disruption, which challenged compliance, as reported by other researchers [26]. This argues for such social disruption to be taken into account when planning how best to protect affected people and control transmission.

Safe burial using plastic bags, lack of burial clothes, and the absence of women in the burial team were described as showing a lack of honour for the deceased. Burials were described as being more compliant to control measures when practices such as community prayer were permitted. In addition, the burial team started to dress in PPE after arrival in the village as now recommended by WHO Guidelines [27]. Additional measures that can be implemented without compromising safe burial, such as including female members in the burial team, and safe alternatives to plastic burial bags, would further enhance community acceptance compliance, and should be included in EVD control guidelines.

The comprehensive design of this study enabled every household in the village to be surveyed, and therefore a number of deaths were captured by our survey that were not identified by MOHS surveillance, EMC, or burial data. All cases and deaths detected were spatially clustered; this is a key finding since traditional methods to estimate mortality rely on cluster sampling approaches, which in this case could have generated either an under- or over-estimation of EVD, depending on whether the limited number of affected households was randomly selected. This is an important element to take into account while trying to benchmark the burden of highly clustered diseases like EVD. Even in a highly affected community, clustering of disease means that household sampling is likely to miss many households unless an appropriate estimate of intra-cluster correlation is available. It is noted that it would not have been feasible to carry out exhaustive studies on the wider population in the middle of the EVD outbreak. In future, we recommend developing alternative methods of sampling to estimate disease and mortality that account for the highly clustered nature of diseases such as EVD.

### Strengths and limitations

A major strength of this study is its mixed methods design, which provides a deeper understanding and explanation of the social reactions to dealing with EVD at community level. Half of the EVD cases in this study were not confirmed by PCR. However, they met the suspected case definition, died, had clear epidemiological links with a confirmed case, and some generated secondary cases, some of which were confirmed EVD. The number of deaths may have been under-reported, as villagers may have feared a penalty for not adhering to the mandatory notification by-law. However, it should also be noted that the study was well perceived by villagers, as demonstrated by the participation of the entire village, their help in documenting the transmission chains, and their willingness to tell the story of the village outbreak. We cannot exclude underestimation of the burden of EVD infection in the village by missing mild or asymptomatic cases. We also collected data on morbidity at the time of the outbreak, and three living people reported history of symptoms compatible with EVD, and a history of exposure, but they were never tested or isolated and thus not included in the analysis. If they were true cases, our EVD mortality may be overestimated, however, when we did include these cases in the analysis it did not change our findings significantly. The true EVD infection rate could be known only via a serological study [28].

Incorrect recall of the timing of deaths may have occurred, but the impact of Ebola makes this less likely, and the use of a local community calendar of events aided recollection of timing. In addition, we validated dates and symptoms for cases admitted to the Kailahun EMC, MOHS surveillance, and buried by the burial team. We were able to rebuild accurate dates for the events of each case we identified, validated across multiple data sources.

For the qualitative part of the study, we acknowledge it was more difficult to definitively link community behaviour change with specific measures or events. Furthermore, we recognise that those are reported perceptions recollected at the time of the outbreak, however, these were consistent among the different people interviewed and suggested a shift in the way the community expressed their ideas of EVD. We acknowledge that perception of changes in the village may have been influenced by the differing roles played by community informants, and in relation to the differing experiences of affected vs. unaffected households.

It is also important to note that our observations were based on a single, high-burden village. Our findings are therefore likely to be generalizable to similar rural settings with high levels of transmission. However, it is possible that the outbreak and response would be different in villages with lower levels of transmission, as experience of the disease was an important driver of behavioural change.

Finally, the main limitation of our qualitative work was that questions regarding burial practices seemed to provoke a limited depth of response in particular among affected households. This may have been because respondents were still affected by their loss.

## Conclusion

In this high-burden village, transmission was maintained by a small number of large households; the outbreak was controlled in this community only after prolonged transmission and a high death toll. A key recommendation emerging from these findings is to ensure that large households and immediate family members are prioritized in control and prevention activities. There is also a need to develop novel sampling methods appropriate for estimating mortality for highly clustered diseases like EVD.

Our findings provide practical information on how future interventions could be implemented more humanely and effectively. We emphasise the following factors: recognising the role of communities for their contribution in controlling outbreaks; identifying community liaison roles which can keep families informed of their relatives’ progress in the EMC; ensuring survivors are engaged to increase community trust to delegate care to EMCs; conveying complex health messages around incubation periods and infectivity clearly to the community; using appropriate alternatives to burial in plastic bags; including women in burial teams; and compensating quarantined households and communities to ensure they can maintain and re-establish livelihoods.

Factors underlying delays in implementing control measures included community belief or otherwise in the presence of EVD, lack of trust, and the toll imposed by interventions such as safe burial procedures and the social disruption of quarantine. Early understanding of social norms and experiences and the ability to link this to localised strategies and adapted health interventions would be essential.

Including these findings in future recommendations for outbreak control policy could help to improve the accuracy of mortality estimates and avoid unnecessary deaths and protracted suffering in future outbreaks.

## Abbreviations

AR%: Attributable risk percent
CFR: Case fatality rate
CI: Confidence intervals
CMR: Crude mortality rate
Ct: Cycle threshold
EMC: Ebola Management Centre
EVD: Ebola virus disease
GPS: Geographic positioning system
HRs: Hazard ratios
IQR: Interquartile range
MoHS: Ministry of Health and Sanitation
MSF: Médecins sans Frontières
PAR%: Population attributable risk percent
PCR: Polymerase chain reaction
PPE: Personal protective equipment
WHO: World Health Organization
